# Adaptation of the comprehensive rheumatologic assessment of frailty (CRAF) as a multidimensional frailty screening tool in patients with rheumatoid arthritis in Vietnam

**DOI:** 10.1186/s41927-024-00394-7

**Published:** 2024-06-07

**Authors:** Trang Huyen Tran, Trang Thi Huong Ta, Lan Thi Ngoc Nguyen, Huyen Thi Thanh Vu, Hung Van Nguyen

**Affiliations:** 1 Department of Internal Medicine, Hanoi Medical University, 01 Ton That Tung, Hanoi, Vietnam; 2 Centre for Rheumatology, Bach Mai Hospital, Hanoi, Vietnam; 3 Department of Geriatrics, Hanoi Medical University, Hanoi, Vietnam; 4 National Geriatric Hospital, Hanoi, Vietnam

**Keywords:** Frailty, Rheumatoid arthritis, Validation study

## Abstract

**Background:**

In recent times, there has been acknowledgment of the prevalence of frailty and pre-frailty among individuals with rheumatoid arthritis (RA). Comprehensive Rheumatologic Assessment of Frailty (CRAF) stands out as a dependable tool grounded in synthesis and clinical judgment. Despite this, a validated Vietnamese rendition of the CRAF is currently unavailable. This study seeks to assess the reliability and validity of the CRAF in a patient with RA in Vietnam.

**Methods:**

A cross-sectional investigation was carried out with 402 patients diagnosed with rheumatoid arthritis, encompassing both inpatients and outpatients at the Centre for Rheumatology at Bach Mai Hospital in Hanoi, Vietnam. CRAF was employed to gauge the extent of frailty. To establish convergent validity, the scores from the CRAF were correlated with those from the Fried phenotype. Discriminant validity was ascertained through the utilization of receiver operating characteristic (ROC) curve analysis. Additionally, a multivariate logistic regression model was applied to evaluate the individual determinants’ relative impact on the CRAF.

**Results:**

In testing for convergent validity, a significant correlation was found between CRAF and Fried phenotype (*p* < 0.001). The discriminatory power of CRAF was higher than those of the Fried phenotype (difference between areas under the ROC curves = 0.947 (95% CI: 0.927–0.967). Variables associated with frailty at the multivariate analysis were comorbitidy, medication intake, BMI, DAS28-CRP, and age (all at *p* < 0.01).

**Conclusion:**

CRAF exhibited strong validity and accurate discrimination. Incorporating frailty assessment into regular rheumatological practices could signify a significant advancement in the care of rheumatoid arthritis.

## Introduction

Rheumatoid arthritis (RA) is a widespread chronic condition characterized by inflammation that predominantly targets the joints, leading to their functional impairment and reduced performance [1, 2]. This limitation in functional capacity can contribute to an increased vulnerability known as frailty [3]. Frailty is a state characterized by a decline in various domains, making individuals become less capable of responding to physical or psychological stressors [4]. The prevalence of frailty among general adult populations affected by RA exhibited a wide range, spanning from 10.1% (as determined by the frailty phenotype) to 36% (as assessed using the Comprehensive Rheumatologic Assessment of Frailty (CRAF), with ‘moderate frailty’ as the threshold) [5]. The incidence of frailty increases as people get older, i.e. 7–10% among people over 65 years old and 20–40% among octogenarians [6].

The presence of frailty in people with RA has been linked to adverse outcomes, including an elevated risk of falls, hospitalizations, and mortality [7, 8]. Studies reported an increased risk of hospitalization among frail RA patients as compared to their non-frail counterparts [9]. Furthermore, frailty in people with RA directly links to increased mortality risk, emphasizing the clinical significance of frailty as a predictor of adverse outcomes within this population [10]. These findings underscore the importance of incorporating frailty assessments into comprehensive care for RA patients, thereby enable healthcare professionals to identify and mitigate frailty-associated risks, ultimately improve the overall health trajectory and outcomes for individuals living with rheumatoid arthritis.

Assessing frailty in RA patients involves the employment of various assessment tools to capture the multifaceted nature of this condition. The Fried phenotype, an extensively used instrument, incorporates such criteria as accidental weight reduction, self-declared fatigue, lack of strength, reduced walking pace, and minimal physical activity. However, this model overlooks cognitive and mood disorders [6]. Other tools include the Clinical Frailty Scale (CFS), offering a comprehensive assessment across various health domains, and the CRAF, specifically tailored for rheumatology patients, considering factors like age, comorbidities, medications, and functional status [11, 12]. Nevertheless, it remains complex, necessitating further psychometric exploration and validation within rheumatological contexts [13]. Consequently, assessing frailty in patients with long-term inflammatory arthropathies presents challenges in routine clinical practice.

Thus, screening, early identification, and strategic intervention for frailty in elderly individuals are crucial aspects of RA treatment. However, there is still a lack of researches on this topic in Vietnam. In recent years, various tools for assessing frailty have emerged, with the Fried phenotype being prominently used in Vietnam’s epidemiological studies to predict clinical outcomes like re-hospitalization, mortality, falls, and fractures. This study aims to validate and implement the CRAF within the context of a low-middle-income country such as Vietnam, specifically tailored for assessing frailty among patients with rheumatoid arthritis. The primary goal is to develop a user-friendly tool can be readily integrated into clinical practice in Vietnam, addressing the unique needs and challenges faced by healthcare providers in this setting.

## Methodology

### Study population and design

This study was a cross-sectional validation analysis that utilized data from a prospective cohort study, involving both inpatients and outpatients at the Centre for Rheumatology of Bach Mai Hospital in Hanoi, Vietnam. Data was collected between March 2023 and January 2024.

The inclusion criteria were people aged ≥ 18 and diagnosed with rheumatoid arthritis according to the ACR/EULAR 2010 criteria, with total score ≥ 6. Patients were required to be able to understand questionnaires, make themselves available for laboratory tests and functional tests as prescribed, and agree to participate in the study. Our research excluded individuals with severe dementia, significant hearing or visual impairments, major functional disabilities, or those requiring contact precautions for multidrug-resistant organisms to prevent issues with communication or cooperation.

### Sample size

We computed an adequate sample size for purpose of obtaining reliable and valid results using a single population proportion formula: n = Z_1−α/2_^2^ x [p x (1-p)/d^2^]. Based on the findings created by Salaffi et al. (2020) [14], we estimated that 35.1% of the rheumatoid arthritis patients have severe and moderate frailty symptoms. With n = the required sample size, Z_1−α/2_ = 1.96 (with α = 0.05 and 95% confidence interval), the required sample size was 350 patients. we successfully interviewed 402 patients with rheumatoid arthritis. All data collected from these 402 interviews were included in the analysis, ensuring comprehensive utilization of the available resources.

### CRAF

CRAF is a newly developed and validated comprehensive index. This index removes the necessity for a calculator and assesses ten health domains: nutritional status, weakness, falls, comorbidity, polypharmacy, social activity, pain, fatigue, physical function, and depression. Each domain’s importance is preset in a specific table. For measuring handgrip strength in the weakness domain, only a dynamometer is needed [15]. Each domain is scored as either 0 or 1, and the final score is derived by averaging the scores across all 10 domains, ranging from 0 (indicating no deficits present) to 1 (indicating all deficits present). Frailty categories are delineated based on Clegg’s criteria: scores from 0 to 0.12 signify the absence of frailty, scores between 0.12 and 0.24 denote mild frailty, scores between 0.24 and 0.36 indicate moderate frailty, while scores exceeding 0.36 signify severe frailty [8].

### Translation of the CRAF into Vietnamese

With Dr. Marco Di Carlo’s permission, we initiated the translation of CRAF into Vietnamese using Brislin’s translation model [16, 17]. Initially, the English CRAF, also known as the source CRAF, was translated by an author of this study and an independent bilingual translator. Two experts then reviewed and compared these translations with the original CRAF to ensure accuracy. Subsequently, back translation was performed by two bilingual primary care physicians unfamiliar with the original CRAF. Finally, a discussion involving bilingual experts along with a geriatric and a rheumatology expert was held to review the back translations against the source CRAF. Any small inconsistencies identified were addressed, leading to consensus among the expert reviewers on the finalized Vietnamese version of CRAF.

### Data analysis and CRAF validation

Descriptive analysis was presented as numbers and proportion for categorical data and mean and standard deviation for continuous variables. Demographic and clinical measures were compared using Mann-Whitney U-test or Kruskal-Wallis test for continuous variables, and chi-square analysis for discontinuous variables.

The prevalence of frailty within the context of CRAF was determined using criteria established by Salaffi et al. [14]. The validity of CRAF was assessed in two ways. First, we examined its convergent validity by analysing the correlation between CRAF scores and those of the Fried phenotype, as well as various clinical measures employed in the study. To quantify these relationships, we utilized the Spearman’s rho correlation coefficient. Correlations at 0.90 and above were considered highly significant, while those falling between 0.70 and 0.89 were regarded as high. Correlations in the range of 0.50 to 0.69 were classified as moderate, while those between 0.26 and 0.49 were seen as low. Correlations at or below 0.25 were considered to indicate minimal or zero correlation [18]. Second, we conducted an analysis of the Receiver Operating Characteristic (ROC) curve as a second step to assess the discriminative accuracy of the Comprehensive Frailty Assessment (CRAF) in distinguishing between frail and non-frail individuals. To quantify this discriminative accuracy, we calculated the Area Under the ROC Curve (AUC). In accordance with Sweets and colleagues, AUC values ranging from 0.50 to 0.70 indicate low accuracy, those between 0.70 and 0.90 are deemed “useful for some purposes,” and values at 0.90 or above reflect high accuracy [19]. The ROC curve analysis also allowed us to identify the optimal cut-off point corresponding to the maximum sum of sensitivity and specificity.

Finally, to gauge the specific influence of individual factors (covariates) like age, gender, RDCI, BMI, HAQ-DI, DAS28-CRP on the CRAF as the dependent variable, a multivariate logistic regression approach was employed. This analysis utilized backward elimination, incorporating variables with initial univariate analysis p-values of 0.1 or less. A significance level *p* < 0.05 was considered statistically significant.

### Other measurements and instruments

Before conducting the main survey, a pilot study involving 20 participants with diverse socioeconomic backgrounds was conducted to assess the validity of the questionnaire content. Minor adjustments to the wording were made based on feedback from participants. The questionnaire comprised the following information:

#### Socioeconomic characteristics

Participants provided self-reported details about their gender, age, marital status, and educational level.

#### Rheumatoid arthritis treatment-related characteristics

The questionnaire was crafted to investigate the presence of comorbidities in patients using RDCI, medication intake, grip strength was assessed with a dynamometer (Jamar Hydraulic Hand Dynamometer 5030 J1, manufactured in the USA), disease activities (DAS28-CRP), BMI was determined by dividing weight by the square of height (kg/m^2^) and was classified into three categories: underweight (< 18.50), normal (18.50–24.99) and overweight (≥ 25.00), and self-care ability.

## RESULT

### Characteristics of the study participants

The traits of the study participants are outlined in Table 1. Briefly, the population is consisted of 402 RA patients, of which 341 are female (84.8%) and 61 are male (15.2%). Over half of participants (59.0%) were diagnosed with RA for 5 years, 18.4% were living with RA for over 10 years. The mean value (SD) of age was 57.08 (12.47) years, and BMI 21.04 (1.96) kg/m^2^.

**Table 1:** Demographic, laboratory, and clinical information for the entire cohort (402 RA patients)

	Mean	Median	SD	IQR
**Age (years)**	57.08	59.00	12.47	16.00
**BMI (kg/m** ^**2**^ **)**	21.04	21.08	1.96	2.50
**RDCI (score, 0–11)**	1.15	1.00	1.06	2.00
**DAS28-CRP (score, 0-9.4)**	4.05	3.95	0.96	1.27
**Medication intake**	2.73	3.00	1.73	2.00
**HAQ-DI (score, 0–3)**	1.34	1.47	0.59	0.91
**CRAF (score, 0–1)**	0.27	0.26	0.11	0.17
**Fried phenotype (score, 0–5)**	1.79	2.00	1.09	2.00

Regarding the disease activity, the mean (SD) of DAS28-CRP was 4.05 (0.96). The mean (SD) HAQ-DI is 1.34 (0.59). Out of 402 subjects enrolled, 322 (80.1%) reported 1 or more medical comorbidities, the mean (SD) of RDCI is 1.15 (1.06). Poly-pharmacy was prevalent in our study group, with 65.2% of participants taking three or more medications daily, and 13.9% receiving five medicines or more per day. The average daily medication intake among participants was 2.73 (SD 1.73; min = 0; max = 8).

The mean (SD) of Fried phenotype was 1.79 (1.09) while the mean (SD) of CRAF was 0.27 (0.11).

**Fig. 1:**
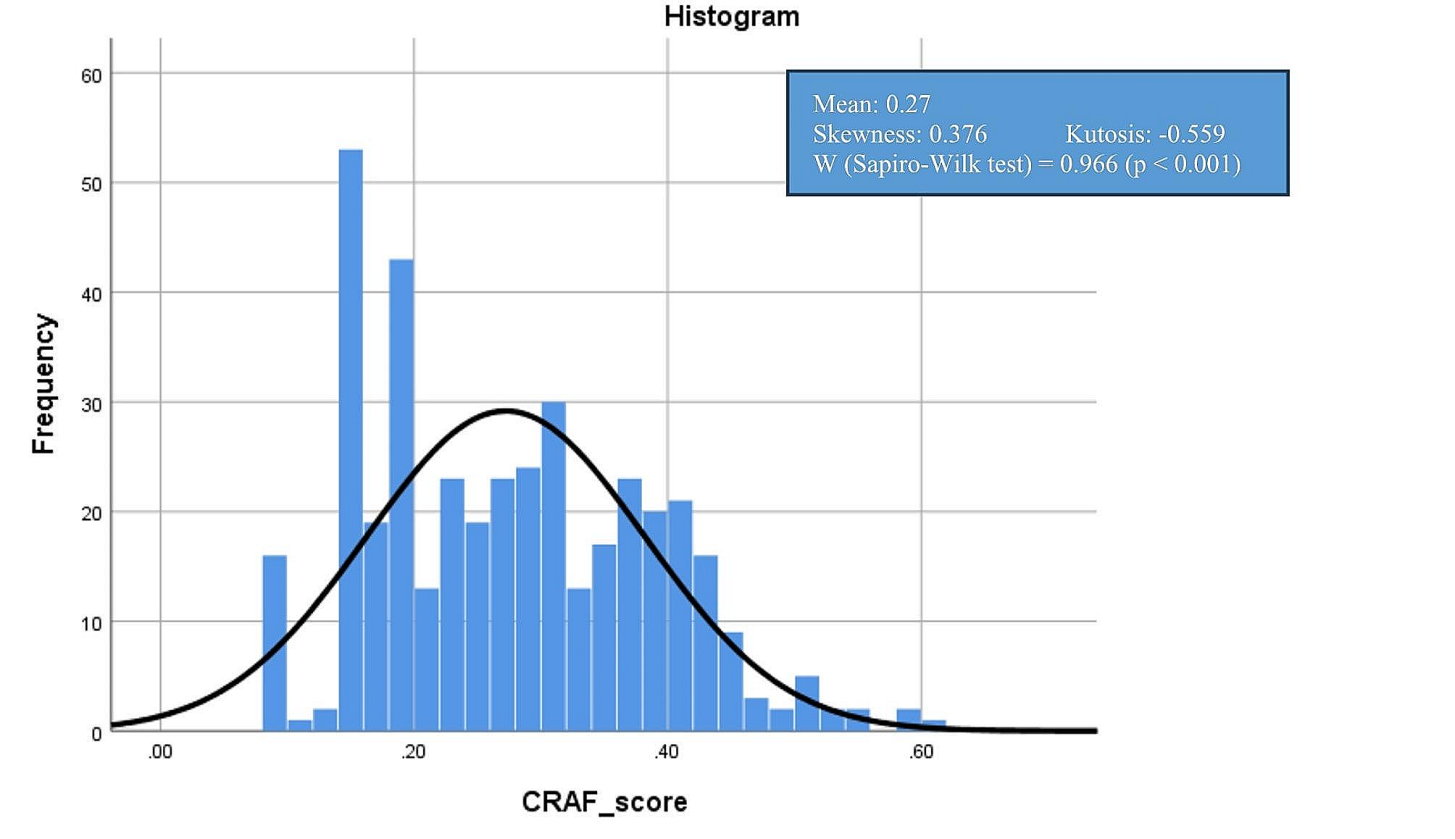
Distribution of CRAF score

### Prevalence of frailty

Figure 1. displays estimates of the central tendency and distribution of CRAF scores, which followed a normal distribution with the result from Sapiro-Wilk test W = 0.966 (*p* < 0.001). The mean and median of CRAF was 0.27 and 0.26 respectively, in accordance with the CRAF criteria, 18 (4.5%) patients were non-frail (normal), 166 (41.3%) were mildly frail, 128 (31.8%) were moderately frail and 90 (22.4%) were severely frail.

### Construct validity of the CRAF

In testing for convergent validity between instruments (Table 2.), it has been found a significant correlation between CRAF and Functional disability HAQ-DI (Coef = 0.659, *p* < 0.001), Fried phenotype FP (Coef = 0.816, *p* < 0.001), and in addition between CRAF and age (Coef = 0.266, *p* < 0.001) and the BMI (Coef = -0.121, *p* = 0.015).

**Table 2:** Convergent validity between instruments: correlation table (Spearman rank correlation coefficient)

	FP	BMI	DAS-28-CRP	HAQ-DI	Age
**CRAF**	0.816**	-0.121*	0.293**	0.659**	0.266**
**FP**	< 0.001	0.015 − 0.091 0.067	< 0.001 0.279** 0.008	< 0.001 0.637** < 0.001	< 0.001 0.170** 0.001
**BMI**			-0.065 0.195	0.017 0.735	-0.021 0.681
**DAS28-CRP**				0.475** < 0.001	0.119* 0.028
**HAQ-DI**					0.240** < 0.001

CRAF: Comprehensive Rheumatologic Assessment of Frailty

DAS28-CRP: Disease Activity Score in 28 joints, C reactive protein version

FP: Fried phenotype

BMI: Body Mass Index

HAQ-DI: Health Assessment Questionnaire Disability Index

**Correlation is significant at 0.01 level (2-tail)

* Correlation is significant at 0.05 level (2-tail)

### ROC curve analysis

**Fig. 2:**
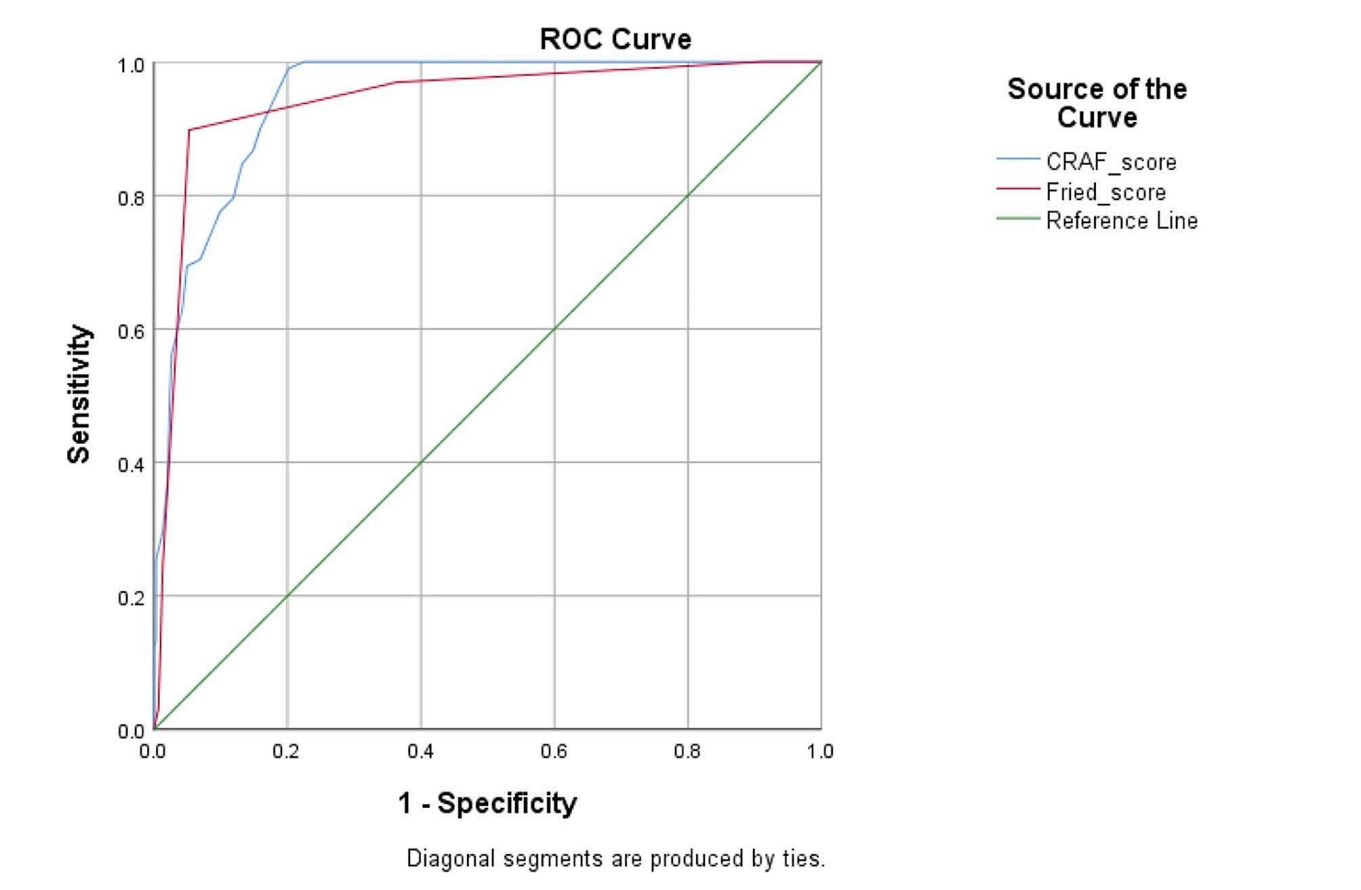
ROC curve analysis of CRAF and Fried score

Figure 2. shows the ROC curve analysis for the CRAF and Fried phenotype which were carried out to assess the ability to discriminate between frail and non-frail patients. The AUC for CRAF was 0.947 (95% CI: 0.927–0.967), *p* < 0.001 and the AUC for Fried phenotype was 0.942 (95% CI: 0.914–0.970), *p* < 0.001. The prognostic cut-off value of the CRAF score was 0.36 (sensitivity, 70.4%; specificity, 93.1%).

### Variables associated with CRAF

Factors associated with frailty in multivariate analysis are listed in Table 3 CRAF scores were significantly associated with RDCI, medication intake, BMI, and DAS28-CRP(*p* < 0.01). The higher CRAF score, the higher age, RDCI, medication intake and DAS28-CRP. The higher CRAF score, the lower BMI.

**Table 3:** Factors associated with frailty in multivariate analysis

	Coefficients	Std. Error	t	Sig.
**(Constant)**	0.128			0.014*
**Age**	0.001	0.000	2.065	0.04*
**RDCI**	0.019	0.005	4.257	0.000**
**Medication intake**	0.029	0.003	10.889	0.000**
**DAS28-CRP**	0.028	0.004	6.375	0.000**
**BMI**	-0.05	0.002	-2.487	0.013*

**Correlation is significant at 0.01 level (2-tail)

* Correlation is significant at 0.05 level (2-tail)

CRAF: Comprehensive Rheumatologic Assessment of Frailty

DAS28-CRP: Disease Activity Score in 28 joints, C reactive protein version

RDCI: Rheumatic Disease Comorbidity Index

BMI: Body Mass Index

## Discussion

In this study, we adapted and translated the CRAF as a tool to assess frailty in patients with RA. This study endeavours to address the void left by the lack of dedicated measurements for RA. The methodology for employing the CRAF was constructed upon established techniques, comprehensively evaluating diverse aspects of frailty in RA patients.

This study found that 22.4% of patients with RA exhibited severe frailty and 31.8% experienced moderate frailty. This prevalence of frailty is greater than in other studies in Vietnam, including studies in the elderly with RA [20–22]. A multi-country study, which involved China, Ghana, India, Mexico, South Africa, and Russia utilizing both frailty phenotype and frailty index criteria showed the lower rates. In particular, the prevalence rates for the former ranged from 8 to 15%, while the latter exhibited a range of 13–56% [23]. The variation observed could be attributed to disparities in the criteria and components employed to evaluate the extent of frailty. Past research has indicated that frailty rates within each community can vary, depending on such factors as definitions, population characteristics, and study methodologies [20, 24]. Given the notably elevated prevalence of frailty among the elderly in the community, regular health screenings are imperative. Such screenings can proactively mitigate the risk of adverse outcomes, including cardiovascular diseases, depression, fractures, falls, hospitalization, and even mortality [19–21].

There is a significant correlation between CRAF score and the Fried phenotype. Many of Vietnamese studies chose to use Fried phenotype as the standard to screen and access frailty among adults, especially the elderly with comorbidity. One benefit of the Fried phenotype is its concise assessment, relying on just five factors (unintentional weight loss, weak grip strength, diminished grip strength, slowness, and minimal physical activity), enabling a relatively quick evaluation [25]. However, the use of CRAF now spans ten areas including nutritional status, weakness, falls, comorbidity, polypharmacy, social activity, pain, fatigue, physical function, and depression thereby improving the precision of screening [26].

Furthermore, the study aligned with many others in terms of the correlation between CRAF and HAQ-DI [27]. Various studies examined the link between frailty and disease activity measures and/or HAQ. They all came to a consensus that frail patients exhibited higher disease activity levels and HAQ scores. This underscores the challenge of assessing outcomes in elderly patients, where the outcomes derived from disease-specific tools might be influenced by the presence of frailty, or patients could appear frail due to elevated disease activity [28].

The study also indicates the relationship between frailty and comorbidity via RDCI. This result was backed up by different studies [29, 30]. Frailty can make individuals more susceptible to chronic diseases, but it can also originate from the presence of multiple coexisting health conditions. In addition, frailty is often associated with various health deficiencies, which suggests the need for treatment with multiple medications specific to the disease [31]. Especially, up to 98% of general elderly experience multimorbidity [32].

Moreover, similarly to our results, polypharmacy is a defined risk for frailty [33]. The profound impact of frailty often compels the extensive use of multiple medications, even when adhering to disease-specific guidelines. Yet, a crucial revelation arises when we consider the individualized, person-centred care of older individuals, highlighting the potential inappropriateness of such medication regimens [34].

Extending beyond individual studies, a body of research across various countries corroborates the relationship between frailty, as assessed by the CRAF, and disease activity measures like DAS28-CRP in RA patients. For instance, a cross-sectional study by Aletaha et al. employing the DAS28, which incorporates tender and swollen joint counts, patient global assessment, erythrocyte sedimentation rate (ESR), or C-reactive protein (CRP) levels, found that higher disease activity was significantly associated with increased frailty levels [35]. These findings are critical as they highlight the need for comprehensive disease management strategies that not only target the reduction of disease activity but also consider the overall functional status and frailty of the patient. By addressing both disease activity and frailty, healthcare providers can offer more holistic care to RA patients, potentially improving outcomes and quality of life.

The assessment of frailty in individuals with RA provides valuable insights for strategic planning of healthcare and social programs. Medically speaking, the term ‘frail’ characterizes patients who possess a diminished ability to adequately respond to external stressors. Elevating the significance of frailty to this extent underscores the need to prioritize the adoption of tools capable of capturing multifaceted risk profiles. This is the essential path to enable the support of care models founded on comprehensive assessments and multidisciplinary intervention plans. Thus, with the comprehensive approach of CRAF, this tool would provide a better view of frailty and multimorbidity and polypharmacy.

Similarly, to many studies, the relationship between CRAF score and age could defined significantly in this study. For instance, a longitudinal cohort study by Rockwood et al. (2011) observed a significant increase in the prevalence of frailty with each decade of life, with individuals aged 80 and above exhibiting a higher likelihood of being frail compared to their younger counterparts [36]. Furthermore, a systematic review and meta-analysis by Collard et al. (2012) found a strong association between age and frailty, emphasizing the progressive nature of frailty as a person ages [37]. The incorporation of comprehensive geriatric assessments, including the use of validated instruments such as the CRAF, enhances our understanding of the relationship between frailty and age, contributing to the development of targeted interventions aimed at mitigating frailty-related adverse outcomes in older adults.

Certain drawbacks of this study need consideration. Firstly, the implementation of language translation means potential language bias cannot be dismissed. Secondly, the cross-sectional design of the study precludes any definitive conclusions about the ability to predict health outcomes related to frailty.

## Conclusion

Our study provided strong evidence that CRAF exhibited strong validity and accurate discrimination. Incorporating frailty assessment into regular rheumatological practices could signify a significant advancement in the care of rheumatoid arthritis.

## Data Availability

The datasets generated and/or analysed during the current study are available from the corresponding author on reasonable request.

## Abbreviations

BMI: Body Mass Index
CFS: Clinical Frailty Scale
CRAF: Comprehensive Rheumatologic Assessment of Frailty
DAS28-CRP: Disease Activity Score in 28 joints, C reactive protein version
FP: Fried Phenotype
HAQ-DI: Health Assessment Questionnaire Disability Index
RA: Rheumatoid Arthritis
RDCI: Rheumatic Disease Comorbidity Index
ROC: Receiver Operating Characteristic
